# Tracking Systemic and Ocular Vitamin A

**DOI:** 10.3390/cells15020163

**Published:** 2026-01-16

**Authors:** Diego Montenegro, Jin Zhao, Hyejin Kim, Sihua Cheng, Janet R. Sparrow

**Affiliations:** 1 Department of Ophthalmology, Columbia University Medical Center, 701 W., 168th St, New York, NY 10032, USA; dm3360@cumc.columbia.edu (D.M.); jz120@cumc.columbia.edu (J.Z.); hjk0609@gmail.com (H.K.); sc5436@cumc.columbia.edu (S.C.); 2 Department of Pathology and Cell Biology, Columbia University Medical Center, 630 W., 168th St, New York, NY 10032, USA

**Keywords:** vitamin A, retina, bisretinoids, retinoids

## Abstract

Vitamin A in the form of 11-*cis*-retinaldehyde is the chromophore essential to vision. Thus, deficiencies in vitamin A necessitate the implementation of vitamin A supplementation. Moreover, some vitamin A is lost from the visual cycle due to random reactions that generate diretinaldehyde (bisretinoid) molecules; the latter are photoreactive and contribute to retinal disease. Here, we measured the systemic and ocular uptake of vitamin A along with bisretinoid as a function of vitamin A availability when supplied in the diet or by weekly i.p. injection in light- and dark-reared mice. Retinyl palmitate delivered as an i.p. bolus served to elevate plasma ROL but an associated increase in ocular 11-*cis*RAL was not observed in light- or dark-reared mice. In dark-reared mice, 11-*cis*RAL was more abundant when retinyl palmitate was provided in chow versus weekly i.p. injection; moreover, by the latter route, retinyl acetate was more effective. Conversely in dark-reared mice given retinyl palmitate by weekly i.p. injection versus chow, ocular atRAL was elevated. Liver atRE was elevated by increased retinyl palmitate in chow; the latter also favored elevated 11-*cis*RAL in dark-reared mice. In cyclic light-reared mice, ocular stores of atRE were increased by i.p. retinyl palmitate. With dark-rearing, there was no difference in bisretinoid (A2E) with retinyl palmitate in chow, nor by weekly i.p. injection; notably, bisretinoid levels were lower in cyclic light-reared mice due to photooxidative loss. In summary, light modulates the ocular retinoid, plasma atROL does not predict ocular levels of retinoid or bisretinoid and atRAL is elevated with sustained darkness.

## 1. Introduction

The vitamin A derivative 11-*cis*-retinaldehyde (11-*cis*RAL) is the light-sensitive chromophore of rod and cone photoreceptor cells. Vitamin A is acquired from the diet as all-*trans*-retinol (retinol) or all-*trans*-retinyl ester (atRE) and is absorbed and stored primarily in the liver as atRE [1]. Vitamin A is delivered to retinal pigment epithelium (RPE) as a retinol-RBP4-TTR complex; binding to the Stra6 receptor (Stimulated retinoic acid 6) [2] enables uptake of retinol by the RPE. Once within the RPE, retinol enters the visual cycle, is stored as atRE and is isomerized and oxidized to 11-*cis*RAL. RBP4 is the specific transport protein for vitamin A (retinol) in blood [3]. Yet, even in the absence of Rbp4, when *Rbp4*^-/-^ mice are born with impaired visual function, 11-*cis*RAL reaches levels that are 60% of wild-type and ERG responses are readily recorded [4,5]. Similarly, with null mutations in human RBP4 [6], patients have undetectable levels of serum retinol but can have good visual acuity (20/20, 20/60) and complain only of night blindness in the second decade of life. These findings are likely explained by alternate delivery mechanisms. For instance, uptake of dietary fat-soluble vitamin A into chylomicrons that form in the intestine after a meal is similar to the handling of neutral lipids (cholesterol and triglycerides). With the remodeling of serum chylomicrons, lipoprotein lipase acts not only on lipid but also hydrolyzes atRE; the product retinol is then taken up by extrahepatic cells [1] such as RPE. AtREs formed in enterocytes are packaged into chylomicrons assembled through the activity of microsomal transfer protein MTP [7]. Accordingly, transgenic mice carrying an RPE-specific knockdown of *Mttp*, the gene encoding MTP, exhibit reduced apolipoprotein B-containing lipoproteins in RPE along with diminished atRE [8]. Dietary retinyl palmitate is enzymatically converted to retinol in the intestinal lumen before absorption by enterocytes of the small intestines.

Retinaldehyde, both the all-*trans*- and 11-*cis*-forms, can also leave the visual cycle by reacting non-enzymatically with phosphatidylethanolamine (PE) (2:1 ratio), one of the phospholipids that dominate photoreceptor outer segments [9]. The vitamin A aldehyde adducts (bisretinoids) thus formed are transferred to RPE and become the lipofuscin of retina. Bisretinoids are transferred to RPE cells within phagocytosed outer segment disks and accumulate as lipofuscin [10,11,12,13].

Bisretinoids form when mice are raised under both cyclic light and continuous darkness [13]. The rate of bisretinoid formation can be modulated by visual cycle kinetics [14,15,16,17]. Thus, limiting delivery of retinol to RPE [18] and reducing the activity of the isomerase RPE65 by a gene variant [14] or compound [13,19,20] reduces bisretinoid formation and protects against the adverse consequences of their accumulation. On the other hand, bisretinoid formation is accelerated in humans and mice deficient in *Abca4* [13,14,21,22,23] and retinol dehydrogenase [24,25,26,27]. Bisretinoids are also increased in mice raised on a high-fat diet [13].

Bisretinoid that has accumulated can also be lost by photooxidation and iron-mediated oxidation [13]. Accordingly, the toxicity of bisretinoids [13] is likely attributable, in good measure, to bisretinoid oxidation products. These light-sensitive compounds photogenerate reactive oxygen species and photodecompose into dicarbonyl- (glyoxal, GO; methylglyoxal, MG) and aldehyde-bearing fragments [13,28] that damage proteins and other macromolecules. qAF levels observed amongst healthy-eye individuals at a given age reflect the balance between formation and loss due to oxidation. We have previously reported that, in wild-type mice, dark-rearing and ocular pigmentation are associated with higher concentrations of bisretinoid in retina due to reduced photodegradative loss [13].

Here, we have studied the relationships amongst modes of delivery and quantities of vitamin A intake and their impact on plasma, liver and ocular retinoid levels. Since vitamin A deficiency may be treated by systemic supplementation, we have included intraperitoneal injection as a means of vitamin A provision.

## 2. Materials and Methods

### 2.1. Mice

Albino BALB/cJ and agouti 129S1/SvImJ mice were purchased from Jackson Laboratories (Bar Harbor, ME, USA) and bred in-house. All mice expressed the leucine-450 variant in Rpe65 [13,14]. Mice were housed under 12 h on–off cyclic lighting with in-cage illuminance of ~30 lux or were dark-reared (with exposure to <1 h/day red light from birth) as previously reported [13]. Both male and female mice were studied. Animals were housed under an ambient temperature of 25 °C. Mice were anesthetized with ketamine and xylazine and pupils were dilated bilaterally (1% tropicamide, 2.5% phenylephrine hydrochloride, Covetrus, Portland, ME) for in vivo imaging. Animal protocols were approved by the Institutional Animal Care and Use Committee of Columbia University (#AC-AABN0559, 22 June 2021; #AC-AACA7703, 6 June 2024). Animals were group-housed in an AAALAC-approved animal facility in accordance with the National Institutes of Health “Guide for the Care and Use of Laboratory Animals” and complied with guidelines set forth by the ARVO Animal Statement for the Use of Animals in Ophthalmic and Vision Research. The numbers of animals were determined based on preliminary experiments, previous related studies and power analyses based on a 0.05 significance level and 0.80 statistical power. All healthy animals were included if they completed the treatment period. Female and male mice were studied and groups of mice were matched by age, genetic background and pigmentation. Mice were euthanized at mid-day and were not fasted.

### 2.2. Diets and Vitamin A Intake

Casein-based vitamin A controlled chows were obtained from Teklad Custom Diets (Madison, WI, USA). Retinyl acetate and palmitate were purchased from Sigma (St. Louis, MO, USA), dissolved in saline and corn oil, respectively, and sterile filtered. To attain vitamin A deficiency, casein-based vitamin A-free chow (TD.86143, Teklad Custom Diets, Madison, WI, USA) was fed to lactating mothers of nursing pups. Pups were nursed by dams on vitamin A-free chow from birth, then weaned on to one of the vitamin A-containing diets (4, 18 and 54 IU retinol/g diet) in the form of retinyl palmitate [29,30], or continued on vitamin A-free chow while receiving weekly vitamin A intraperitoneal (i.p.) injections (112, 504 or 1512 IU, retinyl acetate or palmitate). Other pups were nursed by dams fed breeder chow containing 15 IU/g vitamin A acetate. All diets were 3.8 Kcal/g, 18.8 kcal% protein and 12% fat, purified, bacteria-free, based on AIN-93M diet A [29] and identical except for vitamin A [29]. One IU vitamin A is equivalent to 0.3 µg. Access to food and water was ad libitum.

The recommended daily vitamin A in a mouse is 4 IU/g chow [29]. Here, BALB/cJ mice fed vitamin A at a concentration of 4 IU/g chow acquired 16 IU/day (based on food intake by mice of 4–5 g food/day [29]). Mice were also raised on vitamin A at the upper tolerable level (UTL, 18 IU/g chow; equivalent to 72 IU/d) and 3 times the UTL (54 IU/g chow, equivalent to 216 IU/d). Comparison was made to mice receiving the UTL as a bolus of vitamin A: 112, 504 and 1512 IU by i.p. injection once a week. The UTL was calculated as a 4.5-fold increased concentration of retinoid (relative to sufficient vitamin A) that mimics the fold-change between the human RDA (recommended daily allowance) and the human UTL RDA [31]. Vitamin A delivery by i.p. injection is an approach used in some cases of vitamin A deficiency as it is considered to provide a faster absorption rate.

### 2.3. Fundus Imaging

SW-AF images were acquired from anesthetized mice using 488 nm excitation and a confocal scanning laser ophthalmoscope (Spectralis HRA, Heidelberg Engineering, Heidelberg, Germany), while maintaining cornea hydration (GenTeal gel, Alcon, Geneva, Switzerland) and body temperature. SW-AF intensities (quantitative fundus autofluorescence) were calculated from mean gray levels with normalization to an internal reference as described [13]. High-resolution B-scan images of the retina were acquired by spectral domain optical coherence tomography (SD-OCT) (Bioptigen, Leica Microsystems, Buffalo Grove, IL, USA) as 1.8 mm radial and rectangular volume scans.

### 2.4. Measurement of Retinoids

Retinoids were measured in eyes, liver and plasma. For the quantitation of ocular and liver retinoids [17] (1 mouse eye/sample; 100–250 mg liver/sample), tissues frozen in liquid nitrogen were homogenized in PBS containing 100 mM O-ethylhydroxylamine·HCl, adjusted to pH 6.5 with 4N NaOH and, after the addition of 1 mL acetonitrile, the sample was vortexed. All-*trans*-retinol acetate was added as an internal standard and, after solubilization in hexane and centrifugation, the sample was dried under argon gas and re-dissolved in acetonitrile. The sample was injected into a reverse phase column (CSH C18 column, Waters) for elution in a Waters Acquity UPLC system using gradients of water (A) and acetonitrile (B) containing 0.1% of formic acid as follows: 0–5 min, 60% B; 5–60 min, 60–70% B; 60–70 min, 70–100% B; 70–90 min, 100% B min (flow rate of 0.3 mL/min). Retinal (*O*-ethyl) oximes (11-*cis*RAL and atRAL) were monitored at 360 nm, and atROL and atRE (sum of retinyl linoleate, retinyl palmitate, retinyl oleate and retinyl stearate) were monitored at 320 nm. UV absorbance peaks were identified by comparison with external standards of synthesized retinoids.

For circulating retinoid quantification in plasma, 0.1 mL 0.5 M EDTA was added to the blood and plasma was collected after centrifugation at 4 °C 3000 rpm for 10 min. Subsequently, 1 mL of methanol and 120 µL of *O*-ethylhydroxylamine·HCl was added to 200 µL of the plasma sample and was adjusted to pH 6.5 (final concentration of *O*-ethylhydroxylamine·HCl, 100 mM). Retinoids in plasma were then extracted with hexane (5 mL, 2 times) and dried under argon. The samples were resuspended in acetonitrile (10 µL) and injected into the reverse phase column as described above.

Frozen liver samples were homogenized with glass tissue grinders or glass beads (0.1 and 0.5 mm in diameter) with 2 mL of DPBS (Dulbecco’s phosphate-buffered saline, without CaCl_2_ and MgCl_2_). The tissues were homogenized twice for 15 min each using a Disruptor Genie (Scientific Industries Inc., Springfield, MA, USA). Subsequent derivatization and extraction were performed as previously described [32]. For UPLC analysis, the dried extracts were reconstituted in acetonitrile. For analyses, groups of mice included equal numbers of male and female gender.

### 2.5. Quantitation of Bisretinoids HPLC and UPLC

For the quantitation of bisretinoids (2–4 eyes/sample), mouse eyes were homogenized in DPBS (Dulbecco’s phosphate-buffered saline, without CaCl_2_ and MgCl_2_) and extracted in chloroform/methanol (1:1), or homogenized and extracted in chloroform/methanol (1:1) and, after filtering, the solvent was evaporated as previously described [13]. The extract was re-dissolved in chloroform/methanol and bisretinoids were measured by reverse phase HPLC and UPLC (Waters, Corp, Milford, MA, USA) [14]. An Atlantis dC18 column (3 µm, 4.6 × 150 mm; Waters) was used for reversed-phase HPLC on an Alliance System (Waters Corp., Milford, MA, USA) and gradients of acetonitrile in water with 0.1% trifluoroacetic acid: 75–90% acetonitrile (0–30 min); 90–100% acetonitrile (30–40 min); 100% acetonitrile (40–80 min) with a flow rate of 0.5 mL/min. An Acquity BEH Phenyl Column (1.7 µm, 2.1 × 100 mm; Waters) was used for UPLC on an Acquity UPLC-MS system (Waters), with eluents water–acetonitrile (1:1) with 0.2% formic acid (A) and isopropanol–acetonitrile (9:1) with 0.2% formic acid (B) using a gradient of 0–50 min, 100–55%; 50–110 min, 55–35%. The flow rate was 0.2 mL/min. Absorbance peaks were identified by comparison with external standards and molar quantities per eye were calculated by comparison to standard concentrations determined spectrophotometrically using published extinction coefficients, and normalization to total sample volumes. Two to four (2–4) eyes were combined for bisretinoid measurement, each measurement was expressed as picomoles/eye and, from multiple measurements, mean values were determined.

### 2.6. Histology

Sagittal 5 micron sections were stained with hematoxylin and eosin. Three sections located centrally within the optic nerve head (ONH) were imaged digitally and outer nuclear layer (ONL) width was measured at 200-micron intervals in the sagittal plane. These measurements were then plotted as a function of distance (mm) superior and inferior to the optic nerve head. ONL area was calculated as the measurement interval of 0.2 mm multiplied by the sum of ONL thicknesses in the superior and inferior hemiretina.

### 2.7. Statistical Analysis

GraphPad Prism software (version 10.6.1) was used for analyses; a *p* value < 0.05 was considered significant. Individual data points, mean values and standard deviations (SDs) or standard errors (SEMs) are presented.

## 3. Results

We have undertaken to examine relationships amongst modes of delivery and quantities of vitamin A intake and their links to plasma, liver and ocular retinoid levels. To this end, we varied vitamin A intake delivered in chow ad libitum or by a weekly bolus (intraperitoneal injection, i.p.) and, to understand how light modulates visual cycle retinoid, we studied agouti 129S1/SvImJ mice, albino BALB/cJ mice raised under standard cyclic light and BALB/cJ mice reared in darkness. Bisretinoids form in photoreceptor cells both when mice are raised under a 12/12 light–dark cycle or in continuous darkness [13]. There is also considerable variability in bisretinoid levels even in healthy eyes [13]; not all of the factors determining the variability in the production and accumulation of bisretinoid in retina are understood.

Photooxidative loss of bisretinoid is thwarted under conditions of darkness [13]. Thus, we compared bisretinoid accumulation in mice housed under standard cyclic light and under darkness, the latter allowing us to measure bisretinoid in the absence of photooxidative loss. The inherent short wavelength fundus autofluorescence (SW-AF) that is excited by 488 nm light and imaged by cSLO originates principally from the bisretinoid fluorophores of retina [13,33]. Thus, in addition to bisretinoid quantification by HPLC and UPLC, we also measured qAF intensity as a non-invasive in vivo approach to bisretinoid quantification.

### 3.1. Retinoid Levels in Plasma and Liver

**Figure 1:**
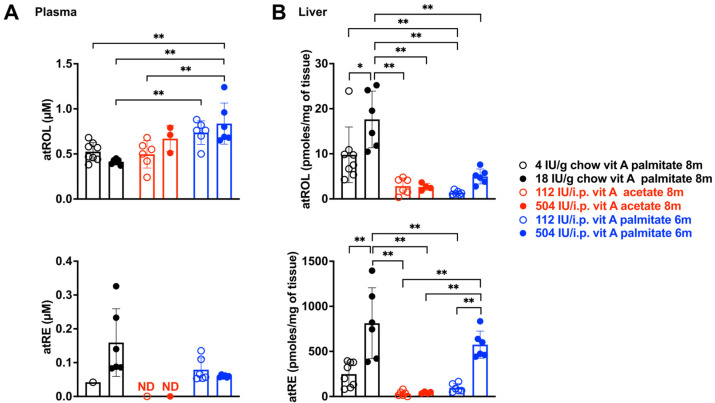
UPLC quantitation of all-*trans*-retinyl ester (atRE) and all-*trans*-retinol (atROL) in plasma (**A**) and liver (**B**). BALB/cJ mice were provided with 4 IU/g or 18 IU vitamin A palmitate per gram chow; 112 IU or 504 IU vitamin A acetate per weekly i.p. injection; and 112 IU or 504 IU vitamin A palmitate per weekly i.p. injection. Mice were aged to 6–8 months. Individual values for each mouse are plotted together with mean ± SD; *n* = 3–8. * *p* < 0.05, ** *p* < 0.01, one-way ANOVA and Sidak’s multiple comparison test. ND (not detected.).

The principal forms of preformed vitamin A used as supplements are retinyl palmitate and retinyl acetate. Retinyl acetate is a synthetic vitamin A derivative synthesized from retinol and acetic acid. Plasma all-*trans*-retinol (atROL) and all-*trans*-retinyl ester (atRE) concentrations were determined by UPLC (Figure 1). As expected, plasma retinoid was primarily present in the form of atROL (Figure 1A), with plasma atRE being only 39% of retinol levels in mice fed vitamin A palmitate at a concentration of 18 IU/g chow (atRE: 0.16 ± 0.1; atROL: 0.41 ± 0.03 μM). In BALB/cJ mice ingesting vitamin A palmitate in chow, there was no difference in plasma retinol whether the dietary content was 4 IU or 18 IU/g chow (*p* > 0.05, two-way ANOVA, Sidak’s multiple comparisons test). However, in a comparison of delivery of vitamin A palmitate in chow versus a weekly i.p. bolus, plasma atROL was higher when vitamin A palmitate was provided as an i.p. injection once a week (18 IU/g chow versus 504 IU i.p. ** *p* < 0.01, one-way ANOVA, Sidak’s multiple comparisons test) (Figure 1A). When plasma atROL and atRE were considered together, the delivery of vitamin A palmitate as an i.p. bolus was even more effective (compare plasma retinoid associated with vitamin A palmitate delivered as 18 IU/g in chow: atRE: 0.16 ± 0.1; atROL: 0.41 ± 0.03 μM; versus delivery as 504 IU i.p.: atRE: 0.06 ± 0.005; atROL: 0.84 ± 0.22 μM).

Plasma retinol is typically used as an indicator of vitamin A status, but, since it is not proportional to the amounts of vitamin A stored in liver [29], we also measured the latter. As expected, when retinoid was provided to BALB/cJ mice in chow or as an i.p. bolus, storage in liver as atRE rather than atROL was the more abundant form (Figure 1B). Levels of atRE in liver were elevated as the quantities of vitamin A palmitate in chow (18 IU versus 4 IU/g chow) or i.p. bolus (504 IU versus 112 IU/weekly i.p. injection) were increased (** *p* < 0.01, one-way ANOVA, Sidak’s multiple comparison test) (Figure 1B).

With an intake of vitamin A palmitate via chow (4 and 18 IU/g chow), atRE storage in liver was higher than with delivery by weekly i.p. injection of vitamin A palmitate; the difference between 18 IU/g chow versus 112 IU by weekly i.p. injection reached statistical significance (** *p* < 0.01, one-way ANOVA, Sidak’s multiple comparison test) (Figure 1B). In mice receiving vitamin A acetate by the weekly i.p. route (504 IU i.p.), liver atRE was lower than with the provision of vitamin A palmitate by weekly i.p. injection (** *p* < 0.01, one-way ANOVA, Sidak’s multiple comparison test). Dark-rearing versus cyclic light-rearing had no effect on levels of retinoid in liver and plasma and thus the data were pooled.

### 3.2. Ocular Retinoid Varies with Vitamin A Intake: Dark-Rearing

**Figure 2:**
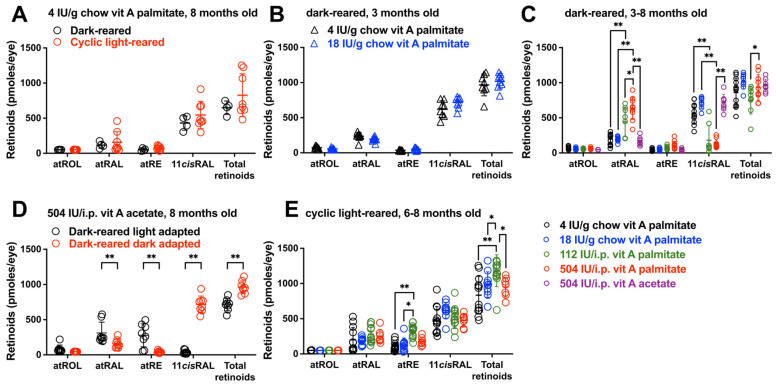
Ocular retinoid in dark- and cyclic light-reared BALB/cJ mice. UPLC quantitation of ocular retinoids all-*trans*-retinol (atROL), all-*trans*-retinal (atRAL), all-*trans*-retinyl ester (atRE), 11-*cis*-retinal (11-*cis*RAL) and the sum of these retinoids (total retinoid). (**A**) Retinoid levels in dark-reared and cyclic light-reared mice fed 4 IU vitamin A palmitate per gram (/gram) chow. Mice were dark-adapted before tissue collection at age 8 months. *n* = 4–8. (**B**) Retinoid levels in dark-reared mice treated with 4 or 18 IU palmitate/g chow. At age 3 months, mice were dark-adapted and tissues were collected. *n* = 8. (**C**) Retinoid levels in dark-reared mice treated with vitamin A palmitate at 4 or 18 IU/g chow and 112 or 504 IU per weekly intraperitoneal (i.p.) injection; or vitamin A acetate at 504 IU per weekly i.p. injection. Mice were dark-reared until age 3–8 months and were dark-adapted before tissue collection. *n* = 8–12. (**D**) Ocular retinoid in dark-reared mice light- or dark-adapted before tissue collection at age 8 months. Mice received vitamin A acetate (504 IU i.p./weekly). *n* = 8. (**E**) Ocular retinoid in cyclic light-reared dark-adapted mice (age 6–8 months). Intake was vitamin A palmitate at 4 or 18 IU/g chow; 112 or 504 IU per weekly intraperitoneal (i.p.) injection. *n* = 8–12. Values per single eye are plotted together with mean ± SD; * *p* < 0.05, ** *p* < 0.01, two-way ANOVA and Sidak’s multiple comparison test.

In the mouse, a quantity of 4 IU/g of vitamin A palmitate in chow is a vitamin A-adequate diet [29]. In BALB/cJ mice that were fed 4 IU/g chow vitamin A in the form of retinyl palmitate and were raised until age 8 months, there were no statistically significant differences in dark-adapted total ocular retinoid levels or in individual ocular retinoids (all-*trans*-retinol (atROL); all-*trans*-retinal (atRAL); all-*trans*-retinyl ester (atRE); 11-*cis*-retinal (11-*cis*RAL) whether the mice were reared in darkness or in cyclic light (*p* > 0.05, two-way ANOVA, Sidak’s multiple comparison test) (Figure 2A). BALB/cJ mice were also provided with vitamin A palmitate at a concentration of 18 IU/g chow, an amount that was 4.5-fold higher than 4 IU but still within tolerable levels [29] (Figure 2B). Amongst 3 month-old dark-reared mice, there was also no difference in (dark-adapted) total ocular retinoids nor 11-*cis*RAL when a dose of vitamin A palmitate at 4 IU/g chow was compared to 18 IU/g chow (*p* > 0.05, two-way ANOVA, Sidak’s multiple comparison test) (Figure 2B). Specifically, ocular retinoids did not vary with whether the dose of palmitate in chow fed to dark-reared mice was 4 vs. 18 IU/g chow.

On the other hand, vitamin A palmitate, when ingested in chow as 4 and 18 IU/g in dark-reared mice, was associated with 11-*cis*RAL levels that were 3- and 5.4-fold greater than with vitamin A palmitate provided as a weekly i.p. injection of 112 or 504 IU (** *p* < 0.01, two-way ANOVA and Sidak’s multiple comparison test) (Figure 2C). There were no differences in 11-*cis*RAL levels when the 112 and 504 i.p. doses were compared (*p* > 0.05, two-way ANOVA, Sidak’s multiple comparison test) (Figure 2C). Interestingly, with weekly vitamin A palmitate i.p. injections (112 and 504 IU), atRAL was higher than with provision in chow (4 and 18 IU/g diet) (112 IU/weekly i.p. injection versus 4 IU/g chow: ** *p* < 0.01; 504 IU/weekly i.p. injection versus 18 IU/g chow; ** *p* < 0.01; two-way ANOVA and Sidak’s multiple comparison test). atRAL levels were also higher under conditions of 504 IU versus 112 IU per weekly i.p. injection (* *p* < 0.05, two-way ANOVA and Sidak’s multiple comparison test). Despite the higher atRAL levels associated with i.p. vitamin A palmitate, total ocular retinoid (sum of atROL, atRAL, atRE, 11-*cis*RAL) was not higher with vitamin A palmitate administered by weekly i.p. injection (112 IU) versus 18 IU/g chow (*p* > 0.05, two-way ANOVA and Sidak’s multiple comparison test) (Figure 2C).

We also compared delivery by i.p. injection of vitamin A palmitate versus vitamin A acetate in dark-reared mice raised until age 6–8 months. Vitamin A acetate provided to BALB/cJ mice as an i.p. injection (504 IU per i.p. weekly injection; equivalent to 18 IU/g chow) was associated with 5.5-fold higher levels of dark-adapted 11-*cis*RAL than vitamin A palmitate (** *p* < 0.01, two-way ANOVA and Sidak’s multiple comparison test) (Figure 2C).

Total analyzed retinoids (sum of atROL, atRAL, atRE, 11-*cis*RAL) levels were not different between the vitamin A palmitate versus vitamin A acetate-treated mice, but atRAL was 4-fold higher in the mice receiving vitamin A palmitate (504 IU/g i.p.) versus vitamin A acetate at the same dose (** *p* < 0.01, two-way ANOVA and Sidak’s multiple comparison test). Ocular storage as atRE was not different amongst mice having an intake of vitamin A acetate (504 IU/weekly i.p. bolus), vitamin A palmitate (504 IU/g i.p. bolus) and 18 IU/g chow (*p* > 0.05, two-way ANOVA and Sidak’s multiple comparison test).

### 3.3. Ocular Retinoid Varies with Vitamin A Intake: Cyclic Light-Rearing

With the cyclic light rearing of BALB/cJ mice (age 8 months), 11-*cis*RAL levels in mice receiving vitamin A palmitate delivery in chow (4 IU, 18 IU) versus a weekly i.p. bolus (112 IU, 504 IU i.p. weekly) were not different as a function of dose or mode of delivery (*p* > 0.05, two-way ANOVA, Sidak’s multiple comparison test) under dark-adapted conditions (Figure 2E). While with dark-rearing atRE did not vary appreciably (Figure 2C), with housing under cyclic light, differences in atRE depended on amount and mode of delivery of vitamin A (i.e., chow versus i.p. injection). Specifically, vitamin A palmitate delivered as 112 IU/i.p. weekly conferred 2-fold and 1.5-fold higher levels of atRE than 4 IU and 18 IU/g chow, respectively (112 IU/weekly i.p. injection versus 4 IU/g chow: ** *p* < 0.01; 112 IU/weekly i.p. injection versus 18 IU/g chow: * *p* < 0.05, two-way ANOVA and Sidak’s multiple comparison test) (Figure 2E) measured after dark-adaptation. Total ocular retinoid was 42% higher when vitamin A palmitate was delivered as an i.p. injection of 112 IU versus 4 IU/g chow and 20% higher as compared to an intake of 18 IU/g chow (112 IU/weekly i.p. injection versus 4 IU/g chow: ** *p* < 0.01; 112 IU/weekly i.p. injection versus 18 IU/g chow; * *p* < 0.05, two-way ANOVA and Sidak’s multiple comparison test) (Figure 2E).

### 3.4. Retinoid Levels in Light- Versus Dark-Adapted Mice

As expected, there was considerable difference in the quantities of 11-*cis*RAL in mice that were dark-adapted versus light-adapted before tissue collection. For instance, in dark-adapted mice provided with 504 i.p. vitamin A acetate, 11-*cis*RAL was 94% lower in mice light-adapted before tissue collection (** *p* < 0.01, two-way ANOVA and Sidak’s multiple comparison test) (Figure 2D). The retinoid sum was also reduced, while atRAL and atRE were increased (** *p* < 0.01, two-way ANOVA and Sidak’s multiple comparison test). These changes are indicative of the photoisomerization of 11-*cis*RAL under conditions of light adaptation; atROL was unchanged.

### 3.5. Effects on SW-AF Intensity and Bisretinoid Levels of Varying Vitamin A Intake with Dark-Rearing Versus Light-Rearing

**Figure 3:**
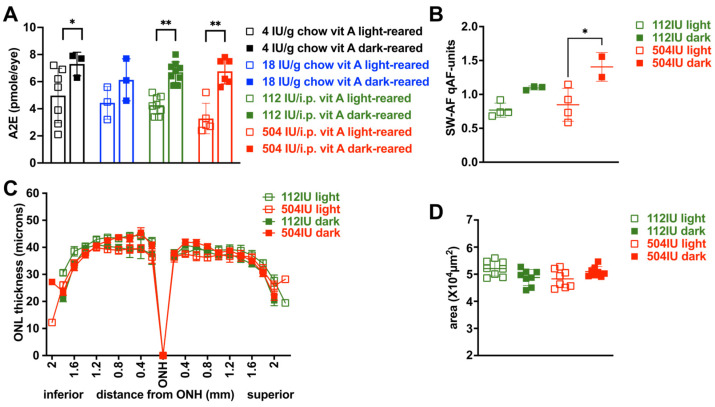
Ocular bisretinoid (A2E and A2-GPE) measured in BALB/cJ mice by HPLC, UPLC and by short wavelength quantitative fundus autofluorescence (SW-AF qAF). (**A**) Intake of vitamin A palmitate provided as 4 or 18 IU/g chow and 112 or 504 IU i.p. weekly. Mice were cyclic light- or dark-reared until age 6 months. Individual values and mean ± SD are plotted; *n* = 3–9 mice. * *p* < 0.05, ** *p* < 0.01, two-way ANOVA and Sidak’s multiple comparison test. (**B**) SW-AF qAF at age 6 months. Individual values and mean ± SD are plotted; *n* = 2–4 mice; * *p* < 0.05, one-way ANOVA and Tukey’s multiple comparison test. (**C**) Outer nuclear layer thickness measured at age 6 months. Intake of 112 IU and 504 IU vitamin A palmitate by weekly i.p. injection in cyclic light and dark-reared mice. (**D**) ONL areas calculated from measurements in (**C**). Values are mean ± SD; *n* = 8 eyes. *p* > 0.05, one-way ANOVA and Tukey’s multiple comparison test.

With an intake of vitamin A palmitate from chow by dark-reared BALB/cJ mice, ocular bisretinoid (A2E) was not increased when the dose of retinoid was increased from 4 IU to 18 IU/g chow, nor when vitamin A palmitate delivery was increased from 112 IU to 504 IU i.p. weekly (*p* > 0.05, two-way ANOVA, Sidak’s multiple comparison test) (Figure 3A). However, with retinoid provided as 4 IU and 18 IU/g chow, bisretinoid levels at age 6 months were lower with cyclic light-rearing versus dark-rearing (* *p* < 0.05, two-way ANOVA, Sidak’s multiple comparison test) (Figure 3A). A2E was also lower in cyclic light-reared versus dark-reared mice provided with 112 and 504 IU (i.p. weekly) (** *p* < 0.01, two-way ANOVA, Sidak’s multiple comparison test). Similarly, with an intake of vitamin A palmitate as weekly i.p. injections (504 IU), measurements of SW-AF intensity (qAF) at age 6 months were lower in mice housed in cyclic light versus darkness (*p* < 0.05, one-way ANOVA, Tukey’s multiple comparison test) (Figure 3B).

### 3.6. Outer Nuclear Layer Thickness in BALB/cJ Mice

To test for a change in photoreceptor cell viability due to the influence of varying vitamin A uptake, we measured ONL thickness. At age 6 months, the BALB/cJ mice did not exhibit differences in ONL thicknesses whether raised in cyclic light or darkness and treated with 504 IU/i.p. weekly injection of vitamin A palmitate. Nor was there a difference in ONL width when the mouse received 112 versus 504 IU/i.p. injection of vitamin A palmitate (Figure 3C). Calculations of ONL area confirmed that the values were not statistically significant (*p* > 0.05, two-way ANOVA, Sidak’s multiple comparison test) (Figure 3D). Similarly, ONL thinning was not evidenced when BALB/cJ were provided with 18 IU and housed in darkness versus cyclic light (ONL area for 18 IU/g dark-reared: 5.67 ± 0.22 × 10^4^ µm^2^; versus ONL area for 18 IU/g cyclic light-reared: 5.26 ± 0.39 × 10^4^ µm^2^; *p* > 0.05, one-way ANOVA, Sidak’s multiple comparison test).

### 3.7. Vitamin A Intake in Cyclic Light-Reared Agouti Mice

**Figure 4:**
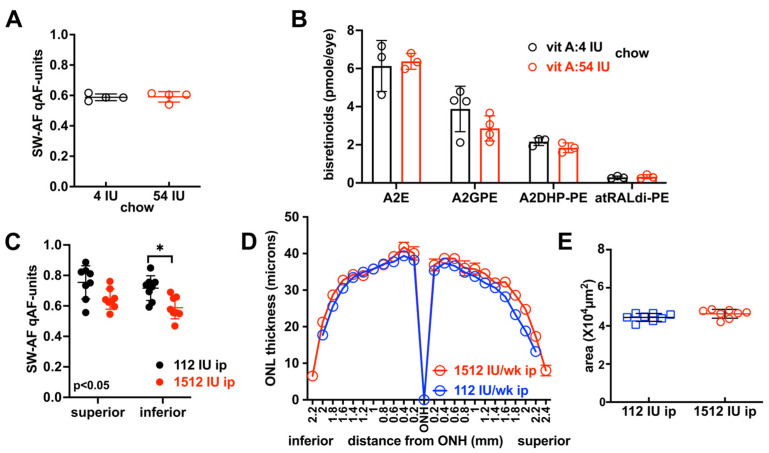
Short wavelength autofluorescence (SW-AF) and bisretinoid quantitation in agouti 129 mice. (**A**) Mice were provided with vitamin A palmitate: 4 IU or 54 IU/g chow. Measurement by quantitative fundus autofluorescence (qAF), age 4 months. Values are mean ± SD, *p* > 0.05, unpaired *t*-test. (**B**) Mice were treated with 4 IU or 54 IU vitamin A palmitate/g chow. Quantitation of bisretinoids by HPLC, age 4 months. A2E is the sum of the all-*trans*- and C13-14-double bond isomers of A2E. Mean ± SD, *p* > 0.05, two-way ANOVA and Sidak’s multiple comparisons test. (**C**) Mice received 112 IU and 1512 IU vitamin A acetate by weekly intraperitoneal (i.p.) injection. Measurement by qAF, age 8 months. Mean ± SD, * *p* < 0.05, one-way ANOVA and Tukey’s multiple comparison test. (**D**) Mice (age 8 months) received 112 IU or 1512 IU vitamin A acetate by weekly i.p. injection. Outer nuclear layer thickness (ONL) plotted as a function of distance from optic nerve head (ONH). Values are mean ± SEM. Mice (age 8 months) received 112 IU and 1512 IU by weekly intraperitoneal (i.p.) injection. (**E**) ONL area calculated from measurements in (**D**). Values are mean ± SD. *p* > 0.05, two-tailed *t*-test. n = 8 eyes. Individual values are plotted (rectangles and circles) together with mean ± SD.

A functional tyrosinase gene in the mouse allows for ocular pigmentation and comparison to the albino under cyclic light-rearing. Agouti 129 mice that were cyclic light-reared and fed vitamin A palmitate in chow at 4 IU versus 54 IU until age 4 months did not exhibit differences in qAF (*p* > 0.05, two-tailed *t*-test) (Figure 4A), the source of which is bisretinoids. Nor were ocular bisretinoid levels significantly different (A2E, A2-GPE, A2-DHP-PE and atRALdi-PE) in these groups of mice at age 4 months (*p* > 0.05, two-way ANOVA, Sidak’s multiple comparison test) (Figure 4B). Agouti 129 mice reared under cyclic light were also given weekly vitamin A acetate by i.p. injection until age 8 months and qAF measures were acquired to compare intensities in superior versus inferior retina. In superior hemiretina, levels were not different when a dose of 112 IU was compared to 1512 IU. However, in inferior retina, the higher dose (1512 IU/i.p. once a week) was associated with lower levels of qAF (* *p* < 0.05, one-way ANOVA Tukey’s multiple comparison test) (Figure 4C). At 8 months of age in agouti 129 mice that received 112 IU/week i.p. (bolus equivalent to 4 IU/g chow) versus 1512 IU/week i.p. (equivalent to 54 IU/g chow), ONL thicknesses were not different (Figure 4D); the latter was confirmed by calculation of ONL area (two-tailed *t*-test, *p* > 0.05) (Figure 4E).

## 4. Discussion

The doses of vitamin A, its form (vitamin A palmitate or vitamin A acetate), the mode of delivery (in chow provided ad libitum, bolus i.p. injection) and whether the mice were dark- versus cyclic light-reared impacted ocular levels of retinoid and bisretinoid in mice.

Vitamin A palmitate and vitamin A acetate both contributed to plasma atROL levels, but the mode of delivery was also relevant. For instance, in mice provided with 112 IU and 504 IU vitamin A palmitate by weekly i.p. injection, plasma retinol levels were higher than in mice provided with 18 IU/g vitamin A palmitate in chow. Moreover, vitamin A palmitate more effectively raised plasma atROL than did vitamin A acetate when both were delivered by i.p. injection weekly. We suggest that vitamin A acetate must be hydrolyzed in order to be transported by RBP4 and delivered to the liver, while palmitate can be readily associated with chylomicrons for transport to liver.

Plasma retinol did not necessarily predict ocular retinoid levels. While, at equivalent concentrations, i.p. vitamin A palmitate was associated with higher levels of plasma atROL than levels acquired by vitamin A palmitate in chow, this difference did not manifest as a difference in total ocular retinoid. Additionally, as a source of 11-*cis*RAL, vitamin A acetate was more efficient. Nor did plasma retinol anticipate bisretinoid levels. For instance, in dark-reared mice, A2E levels were not different whether mice received palmitate as a weekly i.p. injection or ingested in chow.

In liver, atRE was the preferred storage form. Accordingly, atRE content in liver was higher with the delivery of vitamin A palmitate in chow (rather than by i.p. injection) and, in dark-reared mice, delivery in chow also provided more robust levels of 11-*cis*RAL.

In dark-reared mice, 11-*cis*RAL quantities were favored by vitamin A palmitate delivered in chow at both 4 and 18 IU, relative to palmitate delivered at 112 IU and 504 IU i.p. weekly. However, the converse characterized atRAL levels. Specifically, in dark-reared mice, atRAL was increased by the delivery of vitamin A palmitate by weekly i.p. injection versus chow (at equivalent concentrations: 112 IU/i.p. versus 4 IU/g chow; 504 IU/i.p. versus 18 IU/g chow). We suggest that the increase in atRAL occurs due to the isomerization of excess 11-*cis*RAL. The increase in atRAL that we measured is unlikely to consist of free atRAL. Instead, atRAL is likelychaperoned by the formation of a reversible Schiff base adduct with phosphatidylethanolamine (PE) (*N*-retinylidene-PE, NRPE) or with taurine (A1-taurine, A1T) [34,35]. We also note that, in terms of the delivery of retinoid by i.p. weekly injection, 11-*cis*RAL levels are also higher, with acetate at 504 IU i.p. rather than palmitate at 504 IU i.p.

Ocular atRE storage was not optimized by dark-rearing. Specifically, atRE was not different amongst mice provided with 4 IU versus 18 IU and 112 IU versus 504 IU. But, in light-reared mice when atRE was sourced by 112 IU i.p. rather than 4 or 18 IU in chow, atRE levels were elevated.

As mentioned above, in dark-reared BALB/cJ mice, vitamin A palmitate provided in chow enabled higher measures of ocular 11-*cis*RAL and total retinoid. The particularly high levels of atRAL that characterized this intake likely reflects the isomerization of the surplus 11-*cis*RAL that is not needed under dark-rearing. Conversely, in cyclic light-reared mice, vitamin A palmitate delivered by i.p. injection conferred higher total ocular retinoid and increased ocular storage of atRE (as compared to dark-reared mice) that contributed to the elevated total retinoid.

With dark-rearing, there were no differences in A2E levels when mice were fed 4 IU, 18 IU in chow or 112 and 504 IU/i.p. injection weekly (Figure 3A). On the other hand, even though the delivery of retinoid as a bolus of vitamin A acetate (504 IU per weekly i.p. injection) was associated with low atRE liver storage levels, ocular 11-*cis*RAL was higher with an intake of vitamin A acetate (504 IU per weekly i.p. injection) than with vitamin A palmitate at the same dose (504 IU/weekly i.p. injection) in dark-reared mice. Conversely, weekly i.p. delivery of vitamin A palmitate (112 IU or 504 IU per weekly i.p. injection) in dark-reared mice was ineffective in raising 11-*cis*-retinal levels relative to vitamin A palmitate in chow, and was instead associated with elevated all-*trans*-retinal.

The relationships between retinoid levels and bisretinoid were complex. As noted above, bisretinoids form in both darkness and standard cyclic light. We found that bisretinoid levels varied with dark- versus cyclic light-rearing. For instance, in BALB/cJ mice that were reared in darkness, there were no differences in bisretinoid levels whether the mice were provided with 4 or 18 IU vitamin A palmitate in chow or 112 and 504 IU per weekly i.p. injection. Thus, we conclude that the increased availability of dietary retinoid is not necessarily associated with the increased formation of bisretinoid in dark-reared wild-type mice. Notably, in cyclic light-reared mice, A2E levels were lower due to photodegradative loss.

Multiple lines of evidence indicate that the diminished levels of bisretinoid observed here are not attributable to photoreceptor cell loss. Firstly, 11-*cis*RAL levels serve as a marker of photoreceptor cell health; 11-*cis*RAL was not reduced under conditions of elevated vitamin A in BALB/cJ mice (Figure 2C,E). Indeed, the elevation in 11-*cis*RAL also indicated that photoreceptor cells were healthy, since mean 11-*cis*RAL (473 ± 92 pmoles/eye) in 8-month-old mice provided with 504 IU i.p. was not different than 6–8-month-old mice fed 18 IU/chow (619 ± 114 pmoles/eye) in cyclic light-reared mice, or 3-month-old mice fed 18 IU/chow (716 ± 81 pmoles/eye) in dark-reared mice. These robust 11-*cis*RAL levels demonstrated that photoreceptor cells were healthy. Secondly, measurements of ONL width showed that BALB/cJ mice, whether raised in cyclic light or darkness and provided with 504 IU/i.p. weekly, did not exhibit differences in photoreceptor cell densities.

Instead, we conclude that the reduced bisretinoid levels in cyclic light-reared mice versus dark-reared mice was light-related. Agouti 129S1/SvImJ mice that received the highest dose of vitamin A (1512 IU vitamin A palmitate, i.p. once a week) until age 8 months also presented with lower qAF levels in inferior retina, the retina quadrants being exposed to higher retinal illuminance [36,37,38,39,40]. Taken together, these findings indicate that, under environmental light, bisretinoid levels measured at any given time are a product of both bisretinoid that has formed and bisretinoid that has been lost by photodegradation.

Bisretinoid photooxidation and photodegradative loss have been previously documented in mouse models [13], noncellular assays [13] and cellular assays [13]. In in vitro experiments [13], we have observed that the photooxidation of A2E is promoted when the molecules of A2E are located in a milieu that allows the molecules to aggregate. These conditions would include higher concentrations of bisretinoid. The amphiphilic structure of A2E is conducive to aggregation. A2E photooxidation is likely favored by the aggregation of the hydrophobic moieties of A2E and thus increasing the availability of polyene chromophores of A2E for singlet oxygen attack.

We previously studied an individual exhibiting severe vitamin A deficiency due to prior bariatric surgery. SW-AF intensity measured as qAF was profoundly low for his age (197 qAF-units) [41]. After intramuscular injections of vitamin A (300,000 International Units, IU) weekly for 1 month followed by bi-weekly injections for 3 months, qAF increased precipitously (503 qAF-units). Equally important, the topographic distribution of qAF that is marked by higher qAF supero-temporally in healthy eyes was reversed. Specifically, qAF intensity in inferior retina was more pronounced. In the patient, repeated delivery of vitamin A as a bolus likely prompted accelerated bisretinoid formation that greatly exceeded the rate of loss due to photooxidation within the limited time-frame of treatment.

The photooxidation and toxicity initiated by bisretinoids likely also accounts for the ability of light in the absence of melanin to accentuate disease processes in some retinal disorders [40,42,43,44,45,46,47]. In albino mice deficient in Stra6, the receptor and transporter that enables vitamin A uptake by RPE, bisretinoid, was also lost by photodegradation [48]. The photooxidation of bisretinoid generates carbonyl-containing fragments [13,28,49] that form adducts with protein and cross-link. These molecular modifications are detected in aging Bruch’s membrane and drusen [50,51].

We propose that the amount of bisretinoid that forms in retina is higher than previously assumed with loss due to photooxidation obscuring the actual amount. The topographic distribution of SW-AF in healthy eyes exhibits a superior–inferior difference in SW-AF/qAF intensity [13] that may not be established only by rates of formation of bisretinoid but rather by photooxidative loss. Information gleaned from photostasis and sectoral RP indicate that inferior retina (outside central retina) may be exposed to more light than superior retina [36,37,38,39,40].

We previously investigated the effects of supplementation of vitamin A palmitate in mice carrying the p.D190N mutation in rhodopsin (RhoD190N/+; on the C57BL/6J background) and in wild-type (wild-type; C57BL/6J) mice [32]. After 13 months, hepatic and plasma levels of all-*trans*-retinol and all-*trans*-retinyl ester were elevated in RhoD190N/+ mice and wild-type mice fed the vitamin A supplemented diet. While the wild-type mice did not exhibit an increase in ocular retinoids (atROL, atRE and 11-*cis*RAL), these retinoids were markedly increased in the RhoD190N/+ mice. Moreover, the vitamin A-supplemented diet resulted in increased bisretinoid (A2E) in the RhoD190N/+ mice but not in the wild-type mice. Vitamin A supplementation also impaired photoreceptor cell functioning in the RhoD190N/+ mice. The findings in the foregoing study question the rationale of clinical treatments like vitamin A for patients with RP. High-dose vitamin A palmitate supplementation (16,000 IU daily by mouth, 2 months) has been investigated in AMD patients (ClinicalTrials.gov Identifiers: NCT03478865; NCT03478878). That dose is ~4 times the recommended daily allowance (as per FDA). AMD patients exhibited elevated serum vitamin A levels and improved kinetic dark adaptation, although, in patients presenting with reticular pseudodrusen, there was no benefit [52]. Vitamin A supplementation, while once recommended for RP patients [53], is no longer considered to be of benefit to these individuals [54].

The investigation we report here has important implications. We found that both dietary and systemic approaches to vitamin A supplementation are practical and vitamin A palmitate is a more effective source. Secondly, the actual rate of bisretinoid formation in a lifetime may be higher than supposed, with loss due to photooxidation obscuring the true magnitude. And thirdly, the superior–inferior asymmetry in qAF distribution may be established, not just by rates of formation of bisretinoid alone, as previously supposed, but rather by photooxidative loss according to patterns of retinal illuminance.

We note limitations of this study. Mice fed the vitamin A-deficient diet likely produced low levels of retinol by conversion from beta-carotene. This source was not accounted for. Additionally, we assumed that ocular bisretinoid levels observed in dark-reared mice were equivalent to the total amounts of bisretinoid that formed in cyclic light-reared mice.

## 5. Conclusions

Just as light influences retinal metabolism [55], light also adjusts ocular vitamin A levels. For instance, in dark-reared mice, 11-*cis*RAL abundance was advanced by vitamin A delivered in chow rather than by weekly i.p. injection while under standard cyclic light-rearing, an i.p. bolus served to elevate ocular atRE storage. The elevated atRAL that accompanies a bolus of vitamin A under dark-reared conditions likely reflects the isomerization of excess 11-*cis*RAL. Bisretinoids that are measured at any age represent the difference between the lipofuscin pigment that forms versus the pigment that is lost by photobleaching.

## Data Availability

All data in this study are included in the article. Further inquiries can be directed to the corresponding author.

## Abbreviations

The following abbreviations are used in this manuscript:

atRAL	All-*trans*-retinaldehyde
atROL	All-*trans*-retinol
atRE	All-*trans*-retinyl ester
11-*cis*RAL	11-*cis*-retinaldehyde
SW-AF	Short wavelength fundus autofluorescence
qAF	Quantitative fundus autofluorescence
HPLC	High-performance liquid chromatography
UPLC	Ultra-performance liquid chromatography
RBP4	Retinol binding protein 4
RP	Retinitis pigmentosa
AMD	Age-related macular degeneration
